# Thai nurse cohort study: cohort profiles and key findings

**DOI:** 10.1186/s12912-016-0131-0

**Published:** 2016-02-17

**Authors:** Krisada Sawaengdee, Viroj Tangcharoensathien, Tuangtip Theerawit, Petsunee Thungjaroenkul, Wilaiphorn Thinkhamrop, Panuwat Prathumkam, Nathaphop Chaichaya, Kavin Thinkhamrop, Chaiwat Tawarungruang, Bandit Thinkhamrop

**Affiliations:** Thai Nurse Cohort Study (TNCS), International Health Policy Program, Ministry of Public Health, Tiwanon Rd., Nonthaburi, 11000 Thailand; Faculty of Nursing, Chiang Mai University, Chiang Mai, 50200 Thailand; Data Management and Statistical Analysis Center (DAMASAC), Faculty of Public Health, Khon Kaen University, Khon Kaen, 40002 Thailand; Department of Biostatistics and Demography, Faculty of Public Health, Khon Kaen University, Khon Kaen, 40002 Thailand

**Keywords:** Nurse cohort, Workforce shortage, Inequitable distribution, Women’s health

## Abstract

**Background:**

Globally, the nursing profession faces shortages, high turnover, and inequitable distribution. These problems are particularly acute in South East Asia. The present paper describes the design and initial findings of the Thai Nurse Cohort Study (TNCS).

**Methods:**

The TNCS is a longitudinal prospective cohort study comprising multiple age cohorts, initiated in 2009 and expected to run until 2027. Cohorts comprise registered nurses (RN) holding professional licenses granted by the Thailand Nursing and Midwifery Council. Follow-up is at 3-year intervals, with new (younger) TNCS cohorts introduced and older, no-longer eligible members checked out. This maintains the cohort size as representative of the Thai RN population. The first survey round (2009) used a self-administered mailed questionnaire. The second round (2012) provided follow-up of the initial cohort and formed the baseline survey of new entries.

**Results:**

The sampling frame for the first round was 142,699 licensed RN; 50,200 age-stratified participants were randomly selected and mailed the questionnaire, and 18,198 questionnaires were returned owing to incorrect addresses. Of the remaining 32,002 participants, 18,756 (58.6 %) responded (average age 43.7 ± 9.8 years). About 15.4 % (equivalent to 20,000 of the current RN population), reported an intention to leave their nursing career. The second round achieved a follow-up rate of 60.2 %. This round included 3020 participants randomly selected from 6402 new RN (response rate, 38.3 %; mean age 23.1 ± 3.5 years). In this round, 11.2 % reported they intended to leave nursing in the next 2 years.

**Conclusions:**

These two survey rounds have highlighted that Thailand is facing critical nurse shortages. A high rate of nurses expressed an intention to leave the profession; the capacity to replace these potential losses is much lower.

## Background

Globally, the nursing profession is facing shortages, high turnover, and inequitable distribution of the nursing workforce [1, 2]. These are particularly acute problems for South East Asia (SEA), where the nurse-to-population ratio is approximately 2–3 nurses per 1000 population [3], ten times lower than that in Europe although higher than in Sub-Saharan African countries [4]. In SEA, the aging population and high incidence of chronic non-communicable diseases is increasing the demand for nursing and other social care personnel. Moreover, an aging nurse population, lower rates of replenishment by young nurses, and attrition through international migration to high income countries has considerably increased the pressure on the diminishing nurse workforce [5, 6]. The Association of South East Asian Nations (ASEAN) Economic Community (AEC) facilitates the free flow of skilled labor across the AEC, and may have major repercussions on nursing demand and supply in the region [7] where capabilities of health system in attracting and retaining their local staff are unknown [8]. A review suggested that a study investigating these issues conducted locally is essential [9].

Another alarming sign is that aging of nursing profession has been reported in many countries [10–12]. These comprise a large component of the current nurse population and gradually approaching retirement. In addition, the decline in younger women choosing nursing as a career is also observed. However, information about the retirement rate of nurses is limited.

Although numerous studies have identified factors influencing both the supply and demand of nurse labour, most of these studies were conducted in Western countries [5, 6, 13]. Furthermore, most studies investigating nursing crisis are cross-sectional studies. Cohort studies have greater potential to address the crisis as well as the emerging issues in women’s health such as breast cancer, cervical cancer, osteoporotic fractures, postmenopausal illness and its consequence, etc. The study of this kind includes the oldest and the largest nurse cohort study, the Nurse Health Study, initiated in 1976 in the Unites States which focused in cancer and other illnesses [14, 15]. The Danish Nurse Cohort Study (DNCS) established in 1993 investigated working conditions and health including hormone therapy use and risk factors of various health outcomes [16]. The Japan Nurses’ Health Study (JNHS), initiated in 2001, investigated effects of lifestyle and healthcare practices on women’s health [17, 18]. The Nurses and midwives e-Cohort Study, established in 2006, aimed to examine factors associated with recruitment and retention of the nursing and midwifery workforce among nurses in Australia and New Zealand [19]. Recently in 2009, the Nurses’ Early Exit Study (NEXT), a cross-cultural and longitudinal project conducted in ten European countries to analyze the relationship between working conditions and nursing workers’ health [20, 21]. Then Thailand established a nurse cohort study.

A longitudinal cohort study on the health and working life of registered nurses in Thailand was launched in 2009. The Thai Nurse Cohort Study (TNCS) is a 20-year prospective cohort study designed to generate evidence on the workforce dynamics and health status of Thai nurses. Specifically, the TNCS aims to: 1) determine rates, patterns, trends and determinants of job transition, and 2) assess the prevalence, incidence, and long term changes of key health problems among Thai nurses.

It is expected that the TNCS data will clarify a number of key policy and research questions for Thailand, a middle income country; particularly as numerous other studies have investigated nurse populations in high-income countries [14, 22]. The cohort design of the TNCS also has potential to address the dynamics of job transition across public and private sectors, migration, determinants of attrition and retention, and to monitor the impact of policy interventions.

## Methods

### Study design

The TNCS uses a longitudinal design, comprising multiple age cohorts of registered nurses (RN) who hold professional licenses granted by the Thailand Nursing and Midwifery Council (TNC). Participants were selected using age-stratified random sampling using 5-year age groups, from 20 to 64 years (nine age groups in total). The first round, representing the baseline survey, was sampled in September 2009. Data were collected using a self-administered mailed questionnaire.

The TNCS was designed to enroll new members every 3 years, with these new entry cohorts named according to the year of inclusion. New entry cohorts will be sampled from RN newly registered with the TNC and listed in the TNC database. This means the TNCS will have multiple cohorts: the initial cohort collected in 2009, a 2012 new entry cohort, and proposed subsequent new entry cohorts (the 2015, 2018, 2021, 2024, and 2027 cohorts). Cohort members will be followed until 2030, unless they withdraw from participation, are diagnosed with cancer, reach the age of 68 years, or pass away. The second round of the survey, conducted in 2012, served as a follow-up for the initial cohort and provided baseline data for the new entry cohort. The second survey was administered using a mixed mode, either a mailed questionnaire or web-based survey, depending on that member’s preferences. For future surveys, a web-based questionnaire is planned because of the high cost implications of mailed questionnaires.

### Measurements

The questionnaire in the first survey comprised three main sections (Table 1): personal data (birth date, address, marital status, education, income, debt, and other burdens); employment characteristics, job transition since graduation, and job strain questions modified from the Job Content Questionnaire short version (JCQ) [23]; and health status, including the EuroQol five-dimension questionnaire (EQ-5D) [24], the short form of the International Physical Activity Questionnaire (IPAQ) [25], history and current illness, and self-care activities. The follow-up survey repeated the employment and health status sections.

**Table 1:** Parameters and their use in the baseline survey of the Thai Nurse Cohort Study

Parameters	Use
A. Demographic and socio-economics characteristics	
o Thai citizen identification number to be used for ascertainment of morbidity and mortality status in National Health and Death Registry Databases (password protected access)	o Estimating rate of morbidity and mortality for overall and specific to causes or other subgroups o Simultaneously monitoring long-term changes of morbidity and mortality and their determinants o Investigating survival outcome (time to disease, disease progression, dead) o Estimating life expectancy of nurses and its 20 year trends o Determining long-term effect of various factors on mortality and morbidity o Reflecting women health status in Thailand
o Birth place (province and district)	o Characterizing geographically distribution of nurses o Describing work place preference against birth place o Explaining and predicting Job turnover and retention o Testing hypotheses about scholarship or other incentives for working in underserved area
o Birth date or age, sex, marital status	o Investigating their effects on job turnover and health statuses
o Month and year of nursing graduation	o Estimating median survival of working in nursing profession
o Month and year of started working and employment status	o Estimating duration of work o Estimating time gap before employment o Being a baseline employment for investigation of job turnover o Investigating its effect on job turnover and health status
o Institute from which being graduated (public college under the Ministry of Public Health, public university, private educational institute)	o Investigating its effect on retention and workplace preference (urban versus rural, and total survival in profession) o Being a mode for maximizing follow-up rate such as establishing social network among cohort members
o Education (highest level of educational, specialized training, scholarship)	o Determining opportunity of career advancement o Describing distribution of skilled and skill-mix nursing, comparing between urban and rural o Investigating effect of scholarship on work retention and nursing distribution
o Economic status (income, debt, perception of income sufficiency, dependency)	o Investigating its effect on job turnover o Investigating its effect on stress or various health problems as well as health conditions causing the problems
B. Work-life characteristics	
B1. Job transition since graduation	
o Date start working for each roles or employment o Type of work: service and non-service (administrative, teaching) o Leaving nursing for other careers or for un-employ o Study leave o Place of work (public versus private, inside versus outside birthplace)	o Characterizing of job transition pattern over working lifetime o Monitoring of long-term nursing turnover o Describing distribution of nurses between major sectors o Determine pattern of job turnover on intention to stay or leave nursing career o Estimating duration between certain point in time or type of employment and the turnover o Estimating of work-life expectancy (It was 22 years according to the latest estimate in 2007.)
B2. Employment characteristics and working intensity	
o Current employment status (government, private, freelance, others) o Current position (nursing services, administration, research, academic, others) o Current workplace category (hospital, health care center or unit, under study leave, others) o Full time part-time employment, o Work shift arrangement, o Work under night shift, o Working hours per week o Other job in addition to nursing	o Determine current and long-term trend distribution of nurses according to various type of employments, sectors, and employment status o Describing working load as determinants of job turnover and health o Investigating roles of night-evening shift on job turnover and health o Describing work-life balance profile and its role on job turnover and health
B3. Perceived work stresses (21 items, score 1–4 each)	o Estimating proportion of nurses with work stress o Investigating its effect on job turnover o Investigating its effect on various health problems as well as health conditions causing the problems
B4. Workplace violence and occupational injuries	
o Physical harassment, psychological harassment, from colleagues, patient and their relatives	o Estimating proportion of nurses facing workplace violence o Investigating its effect on job turnover
o Occupation injuries: specify: cut wound, needle stick, chemical and radiation injuries, o Hospital acquired infections e.g. TB, Hepatitis, HIV/AIDS,	o Estimating rate and trend of occupational injuries o Investigating its effect on job turnover
B5. Intension to leave and to return to nursing career	
o Intention to leave nursing profession and the reasons for those currently working in nursing profession	o Estimating probability to leave and predicted duration before quit o Investigating its effect on job turnover
o Intention and enabling conditions to return to nursing profession for those currently not engaged in nursing profession	o Estimating probability of return to nursing professions, list of potential policy intervention on retention o Investigating its effect on job turnover
C. Health status and related information	
C1. Anthropometrics, daily activities, and sleeping	
o Current anthropometric measurements: body weight, height, waist and hip circumference	o Investigating its effect on job turnover o Investigating its effect on stress or various health problems as well as health conditions causing the problems
o Intensity of daily physical activities (duration of heavy, moderate, walk, sit activities) o Physical activities at leisure	o Estimating prevalence of regular physical activities, compared to general population o Investigating roles of physical, diets and cholesterol on various chronic diseases and metabolic syndromes
o Average duration of sleeping per day and frequency of sleeping difficulties	o Investigating its effect on job turnover o Investigating its effect on stress or various health problems as well as health conditions causing the problems
C2. History and current illness, and self-care activities	
o Family members who had history of illness being diagnosed by medical doctor	o Investigating its effect on job turnover o Investigating its effect on stress or various health problems as well as health conditions causing the problems
o The respondent’s history of illness being diagnosed by medical doctor and whether or not currently under treatment	o Estimating incidence / prevalence and type of chronic illnesses
o Annual physical checkup being done and their results with regard to breast self examination, cervical cancer screening, chest film, blood examination, lipid measurements, etc.)	o Describing health awareness o Estimating proportion of nurses who had certain problems regarding various laboratory findings, health measurements, or diseases
o Health care services being utilized by the respondent during the last 12 months	o Investigating health profiles
o Hospitalization during the last 12 months	o Investigating health profiles
o Medication used during the last 30 days	o Investigating health profiles
o Self assessment of health in the last 30 days based on EuroQol five-dimension questionnaire (EQ-5D)	o Estimating level of health status according to self-perceptions
o Reproductive health for female respondents (pregnancies, delivery, and family planning methods)	o Estimating total fertility rate among nurse, infertility, prevalence and profile

### Sample size calculation

A sample size of 30,000 was initially planned, based on the aim of detecting any events with a proportion as low as 1 % with +/−0.1 % precision (i.e., it offered a relative precision of +/−10 % with a two-sided 95 % confidence level). We also allowed 40 % for expected non-responses because of invalid contact addresses, refusal to participate, and loss to follow-up; this excess was slightly better than that used in a study involving nurses in Japan [26]. Therefore, the sample size was inflated to 50,000. However, administrative reasons meant that the actual number of questionnaires mailed out was 50,200.

### Sampling techniques

TNCS participants were classified into two categories: the initial cohort and the new entry cohort.

The initial cohort comprised RN who were randomly selected from the TNC database, who signed a consent form, and who responded to the first-wave questionnaire. The TNC database used as the sampling frame included all RN who held nursing licenses granted by the TNC as of 2008, with a total of 142,699 RN forming the sampling frame. We then applied an age-stratified random sampling technique for sample selection. First, RN aged 20–64 years were stratified into nine age groups based on 5-year intervals (< 25, 25–29, 30–34, 35–39, 40–44, 45–49, 50–54, 55–59, and 60–64 years). Based on the sample size of 50,000, the average size of each age group was calculated as 5556 nurses. However, as the oldest two age groups (55–59, and 60–64 years) were smaller than the average size, we selected all members of these groups. Participants from the remaining seven age groups were randomly selected. First, a set of random numbers were generated using Stata statistical software (StataCorp, College Station, TX). This assigned a random number to each nurse’s Citizen Identification Number (CID). CID were then sorted according to the assigned random numbers. Nurses in each age group were listed in the sorted order. Finally, nurses were selected consecutively, starting from the first order, until the specified size was achieved. The sample size was equal for all seven age groups. For administrative reasons, a total of 50,200 nurses were eventually selected for the initial cohort.

The new entry cohort included all RN who held nursing licenses granted by the TNC after 2008 and were not part of the sampling frame of the initial cohort. From a total of 6402 RN in this group, 1600 (25 %) were randomly selected. The selection methods as used for the initial cohort were applied.

### Statistical methods

We provided the probability of responses as the inverse proportion to the probability of responses to be used in subsequent papers for weighting in statistical analyses. To describe the cohort profile, we presented the results as number and percentage for categorical variables, and mean and standard deviation (SD) for continuous variables. These descriptive statistics were used for demographic characteristics, employment history and career advancement, current employment status, intention to leave the nursing profession within the next 2 years, and quality of life based on the EuroQol five-dimensions, in both the initial and the new entry cohorts. All analyses were performed using STATA version 13 (StataCorp).

### Ethics and good clinical practice

The present study was conducted according to the principles of Good Clinical Practice (International Conference on Harmonization Tripartite Guideline for Good Clinical Practice), the Declaration of Helsinki, and national laws and regulations for clinical studies. The TNCS was approved by the Thai Ministry of Public Health Ethics Committee for Human Research. All participants signed a consent form for participation as a cohort member of the TNCS.

## Results

### Questionnaire responses and recommended weighting for statistical analysis

**Fig. 1:**
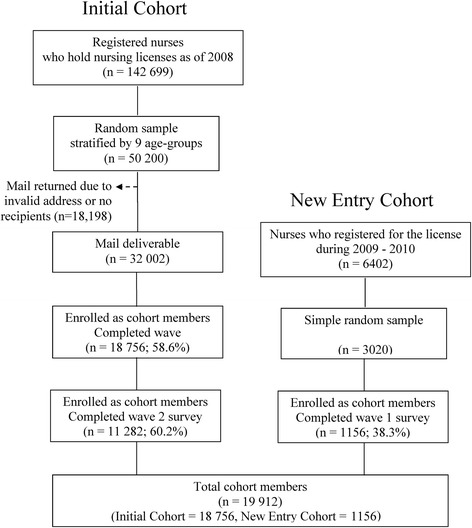
Population, sample, and respondents of the baseline survey of the Thai Nurse Cohort Study

In total, 50,200 questionnaires were sent via postal mail for the first survey wave, 18,198 (36.3 %) were returned owing to invalid addresses or no recipient (Fig. 1). There were 18,756 questionnaires returned with signed and dated consent forms. This gave a response rate of 58.6 % for the 32,002 deliverable questionnaires, assuming the questionnaires were received. The first 10,000 responses were received within 2 months of the mail out.

Although the overall response rate for the first survey was 58.6 % (Fig. 1), the three younger age groups had the lowest response rates (see Table 2 for the probability of responses). For the new entry cohort, the weight was determined based on the inverse proportion of responses, which was 2.61 (3020/1156).

**Table 2:** Recommended weight for data analysis of the initial cohort of the Thai Nurse Cohort Study

Age group	Population	Respondents	Probability	Weight
Lower than 25	9352	747	0.0799	12.5194
25–29	22 664	1308	0.0577	17.3272
30–34	32 807	1760	0.0536	18.6403
35–39	24 400	2690	0.1102	9.0706
40–44	21 760	3155	0.1450	6.8970
45–49	17 640	3584	0.2032	4.9219
50–54	7827	2971	0.3796	2.6345
55–59	4572	2043	0.4469	2.2379
60 or greater	1677	498	0.2970	3.3675
Total	142 699	18 756		

The second round, conducted in August 2012, served as a follow-up for the initial cohort and the baseline survey of the new entry cohort. The follow-up rate for the initial cohort was 60.2 % (11,282 of 18,756 RN who responded in the first round). In the second round, RN who were registered with the TNC in 2009 and 2010, and who were aged between 23–27 and 22–26 years, respectively, formed the sampling frame of the new cohort. Nurses in the new entry cohort were aged 20–24 years when the sampling frame for first round of the study was established. Of the 7050 newly registered RN, 648 were excluded as their age exceeded the required age range. Of the remaining 6402 new RN, 25 % were identified as the final sample size. To allow for 47 % of non-responses based on the previous round survey, a sample size of 3020 was required. Of these, 1156 responded and were enrolled as new entry cohort members. The response rate for the new entry cohort was 38.3 % (Fig. 1).

### Demographic characteristics

The majority of the initial cohort members were female (96.7 %), married (60.8 %), lived in the central region of the country (38.7 %), and had attained a bachelor’s degree (57.2 %) (Table 3). About half (48.5 %) were aged older than 44 years. The average age was 43.7 ± 9.7 years. The average age of the new entry cohort was 23.1 ± 3.5 years, and a much higher proportion were single (82.3 %).

**Table 3:** Demographic characteristics of first-wave survey respondents, presented as number and percentage unless indicated otherwise

Characteristics	Initial Cohort (*n* = 18 756)		New Entry Cohort (*n* = 1156)	
	*n*	(%)	*n*	(%)
Sex				
Male	625	(3.3)	75	(6.8)
Female	18 013	(96.7)	1029	(93.2)
Age (years)				
Lower than 25	747	(4.0)	1119	(96.8)
25–44	8913	(47.5)	24	(2.1)
45 or greater	9096	(48.5)	13	(1.1)
Mean (Standard deviation)		43.2 (9.8)		23.1 (3.5)
Marital status				
Single	5764	(30.9)	950	(82.3)
Married	11 354	(60.8)	192	(16.6)
Widowed	533	(2.9)	6	(0.5)
Divorced	880	(4.7)	3	(0.3)
Separated	152	(0.7)	3	(0.3)
Region of workplace				
North	2234	(12.7)	71	(16.6)
Northeast	4060	(23.0)	96	(22.5)
Central	6828	(38.7)	140	(32.8)
East	1318	(7.5)	28	(6.6)
West	953	(5.3)	24	(5.6)
South	2258	(12.8)	68	(15.9)
Current and highest educational attainment				
Certificate equivalent to bachelor’s degree	4289	(23.4)	24	(2.1)
Bachelor’s degree	10 474	(57.2)	1123	(97.2)
Master’s degree	3170	(17.3)	8	(0.7)
Doctoral degree	154	(0.8)	0	(0)
Other	210	(1.3)	0	(0)

### Employment history and career advancement

A majority of the initial cohort (78.0 %) graduated from nursing colleges owned by the Thai Ministry of Public Health; 79.5 % had been serving mandatory nursing service in public sector after graduation; and 63.1 % were employed as RN when they started working, while the remaining nurses started their career as a Technical Nurse (diploma level) (Table 4). Only 7.9 % had graduated from a public university. The new entry cohort showed similar pattern in their employment history. Around 5833 (31.1 %) of the initial cohort had a diploma-level education at first employment, 75.6 % of these were trained and upgraded (67.1 % to a bachelor’s degree, 8.5 % to a master’s degree, and 0.03 % a doctoral degree). The entire new entry cohort held a bachelor’s degree at their first employment.

**Table 4:** Employment history and career advancement of the first-wave of the Thai Nurse Cohort Study

Characteristics	Initial Cohort (*n* = 18 756)		New Entry Cohort (*n* = 1156)	
	*n*	(%)	*n*	(%)
Institute from which graduated				
Public university	1215	(7.9)	0	(0)
Nursing colleges under Ministry of Public Health	11 964	(78.0)	651	(77.8)
Other nursing colleges	1590	(10.3)	142	(17.0)
Private institutes	574	(3.8)	44	(5.2)
Having been under contracted scholarship				
Yes	14 408	(79.5)	712	(65.9)
No	3712	(20.5)	368	(34.1)
Employment upon started working				
Undergraduate level				
Public health worker	623	(3.4)	4	(0.3)
Assistant nurse	1152	(6.2)	0	(0)
Technical nurse	4220	(22.7)	4	(0.3)
Graduate level				
Registered nurse	11 739	(63.1)	1103	(96.5)
Nurse lecturer	190	(1.0)	22	(1.9)
Others	687	(3.6)	10	(1.0)
Career advancement among initial cohorts who started working at undergraduate level (*n* = 5833)				
Remain unchanged	1365	(23.4)		
Upgraded to Bachelor degree	3915	(67.1)		
Upgraded to Master degree	497	(8.5)		
Upgraded to Doctoral level	2	(0.03)		
Others	54	(0.9)		

### Current employment status

A majority of the initial cohort were engaged in nursing services (61.2 %), and 21.0 % were mid-level nursing administrators (Table 5). Around 80.8 % were civil servants and 79.0 % worked in hospitals. Only 9.0 % worked in health centers or private clinics. Again, a similar pattern of current employment was found in the new entry cohort.

**Table 5:** Current employment status of the first-wave of the Thai Nurse Cohort Study

Current employment characteristics	Initial Cohort (*n* = 18 756)		New Entry Cohort (*n* = 1156)	
	*n*	(%)	*n*	(%)
Employment category				
Direct care nurse	11 320	(61.2)	1094	(95.4)
Nurse lecturer	554	(3.0)	28	(2.4)
Middle level administrator (Head ward, Unit manager, Chair of the department)	3886	(21.0)	2	(0.2)
High level administrator (Director, Dean of the faculty or equivalent)	548	(2.9)	0	(0)
Research nurse	489	(2.6)	3	(0.3)
Unemployed	311	(1.7)	16	(1.4)
Others	1398	(7.6)	4	(0.3)
Main career				
Civil servant	15 032	(80.8)	228	(19.9)
Government officer	702	(3.8)	558	(48.6)
State enterprise staff	93	(0.5)	17	(1.5)
Private sector staff	1415	(7.6)	329	(28.7)
Freelance	177	(1.0)	1	(0.1)
Others	1183	(6.3)	14	(1.2)
Workplace				
Hospital	14 088	(79.0)	1013	(91.8)
Health center, clinic	1600	(9.0)	45	(4.1)
Infirmary in school or other organization	98	(0.6)	1	(0.1)
Nursing school	592	(3.3)	25	(2.3)
Non-health care service office	498	(2.8)	1	(0.1)
Others	959	(5.3)	18	(1.6)

### Intention to leave nursing career

Of the 16,470 nurses in the initial cohort who were working in the nursing profession at the time of the baseline survey, 2306 (15.4 %) stated that they intended to leave their nursing career. This proportion was slightly lower in the new entry cohort, with 11.2 % indicating an intention to leave the profession in the next two years (Table 6). The number of nurses intending to leave the profession increased slightly with age. More research nurses reported an intention to leave, (16.7 %) compared with other employment categories. In the initial cohort, 18.0 % of nurses who worked in research areas and 20.4 % who worked in the private sector reported an intention to leave the profession.

**Table 6:** Intention to leave the nursing profession within the next 2 years, as reported in the Thai Nurse Cohort Study

Subgroups of registered nurse	Initial Cohort				New Entry Cohort (*n* = 1156)	
	Wave 1 (*n* = 18 756)		Wave 2 (*n* = 11 282)			
	Number	% Intent to leave	Number	% Intent to leave	Number	% Intent to leave
Overall	18109	(15.4)	10 762	(5.5)	1127	(11.2)
Age group						
Lower than 25	743	(10.6)	680	(5.4)	1091	(11.2)
25–29	1289	(13.8)	808	(6.2)	8	(0)
30–34	1735	(13.7)	942	(6.3)	5	(0)
35–39	2652	(15.5)	1340	(5.2)	7	(14.3)
40–44	3108	(15.9)	2590	(5.5)	3	(33.3)
45–49	3503	(16.0)	1669	(4.9)	6	(16.7)
50–54	2849	(16.0)	1439	(6.3)	5	(0)
55–59	1915	(15.8)	991	(5.2)	2	(50.0)
60 or greater	315	(18.4)	303	(4.0)	0	(0)
Employment category						
Direct care nurse	11 134	(15.2)	6756	(6.0)	1081	(11.6)
Nurse lecturer	551	(12.7)	289	(5.9)	26	(3.9)
Middle level administrator	3767	(15.2)	2760	(4.9)	2	(0)
High level administrator	534	(15.0)	416	(3.4)	0	(0)
Research nurse	484	(18.0)	254	(2.8)	3	(0)
Main career						
Civil servant	14 715	(15.2)	9158	(5.5)	223	(11.7)
Government officer	692	(11.3)	566	(4.8)	551	(11.4)
State enterprise staff	92	(16.3)	117	(6.8)	16	(12.5)
Private sector staff	1392	(20.4)	707	(6.7)	326	(10.7)
Freelance	150	(12.0)	91	(3.3)	1	(0)
Others	956	(12.8)	84	(1.2)	4	(0)

### Quality of life, health status

Overall, the quality of life of Thai RN, based on EQ-5D scores, was good, with an overall mean score of 0.758 ± 0.191 for the initial cohort and 0.751 ± 0.178 for the new entry cohort (Table 7). In the initial cohort, the mean quality-of-life score remained unchanged at follow-up (3 years after the baseline survey) (mean = 0.764 ± 0.193). Across the EQ-5D dimensions, more than half of the Thai RN reported some degree of pain or discomfort, and about one third reported anxiety or depression.

**Table 7:** Quality of life based on the EuroQol five-dimensions in the Thai Nurse Cohort Study, presented as number and percentage unless indicated otherwise

Subgroups of registered nurse	Initial Cohort				New Entry Cohort (*n* = 1156)	
	Wave 1 (*n* = 18 756)		Wave 2 (*n* = 11 282)			
	*n*	(%)	*n*	(%)	*n*	(%)
Mobility						
I have no problems in walking about	14 180	(76.0)	8392	(74.9)	944	(82.4)
I have some problems in walking about	4445	(23.9)	2807	(25.0)	199	(17.4)
I am confined to bed	25	(0. 1)	9	(0.1)	2	(0.2)
Self-Care						
I have no problems with self-care	18 266	(97.9)	10 923	(97.4)	1120	(98.1)
I have some problems washing or dressing myself	385	(2.1)	279	(2.5)	22	(1.9)
I am unable to wash or dress myself	7	(0.0)	8	(0.1)	0	(0)
Usual Activities						
I have no problems with performing my usual activities	15 439	(82.8)	9470	(84.5)	922	(80.6)
I have some problems with performing my usual activities	3130	(16.8)	1696	(15.1)	220	(19.2)
I am unable to perform my usual activities	88	(0.4)	39	(0.4)	2	(0.2)
Pain/Discomfort						
I have no pain or discomfort	8347	(44.8)	5075	(45.3)	445	(38.9)
I have moderate pain or discomfort	9910	(53.2)	5858	(52.3)	676	(59.2)
I have extreme pain or discomfort	389	(2.0)	265	(2.4)	22	(1.9)
Anxiety/Depression						
I am not anxious or depressed	11 210	(60.2)	7475	(66.8)	683	(59.8)
I am moderately anxious or depressed	7135	(38.3)	3599	(32.1)	436	(38.1)
I am extremely anxious or depressed	283	(1.5)	124	(1.1)	24	(2.1)
Mean (standard deviation) overall score	0.758	(0.191)	0.764	(0.193)	0.751	(0.178)

## Discussion

The TNCS was established to help address the Thai nursing workforce crisis, but may also contribute to the understanding of the health of Thai women engaged in a specific profession. To the best of our knowledge, this is the first cohort study with nurses in SEA. Our findings contribute evidence from a middle-income country, where the socioeconomic profile, environment, and health behaviors differ from those of high-income countries. Understanding long-term nursing workforce dynamics and changes in women’s health is essential for relevant policy formulation. The TNCS was also established at a time (2009) of significant change, with the integration of 10 ASEAN countries into a single economic community, the AEC. This means that the TNCS can monitor the corresponding changes in the Thai nursing workforce, including the impact of the free flow of the nurse workforce in the AEC on nursing mobility and shortages, and contribute to the introduction of timely workforce policy. The present paper was prepared during the preparations for the third TNCS survey round (2015), which will yield further information on the impact of the AEC.

In addition, the nine age-groups covered by the initial TNCS cohort will contribute to the understanding of job transition dynamics; while the regular replenishment of the cohort with younger age groups will contribute to a timely understanding of the work and life dynamics of the younger professional group, allowing new questions to be addressed. This design has many advantages over conventional single-age cohort studies, as highlighted in previous research [27].

The baseline survey response rate for the initial cohort (58.6 %) was similar to or greater than some other large studies conducted among the nursing profession [14, 18, 22]. However, we remain concerned about the lower response rate (38.3 %) for the new entry cohort, and more effort may be necessary to ensure a more favorable response rate in future.

A steering committee including key partners and stakeholders provided advisory support to the TNCS secretariat. These include 1) President of the Thailand Nursing and Midwifery Council (Chair), 2) Director, the International Health Policy Program (Vice Chair), 3) President, Thai Nurse Association, 4) President, Consortium of Dean of Nursing Schools, 5) Director, Bureau of Policy and Strategy, Ministry of Public Health, 6) Director, Nursing Systems Research Institute, Thailand Nursing and Midwifery Council, 7) Director, Prabomarajchanok Institute for Health Workforce Development, Ministry of Public Health, 8) Director, Health Systems Research Institute, 9) Director, HRH Research and Development Office, 10) Director, Bureau of Nursing, Ministry of Public Health, 11) Representative from the National Health Security Office, and 12) Representative from the National Health Commission. The Steering Committee is responsible for oversight and support of the work of TNCS, in line with the specified goals and objectives.

Maintaining a professional cohort with a high rate of follow-up responses and low costs was challenging. We designed the TNCS as part of the existing routine data collection system. First, updated postal addresses and other essential follow-up data including job transition and health status were fully embedded in a form that is completed by all RN during the 5-yearly process of professional license renewal, as legally required based on Continued Nursing Education credits. This form is mandatory and was uploaded to the TNC web-based registration system. The professional licensing renewal requirement enables the sustainability of the cohort, maximizes the follow-up rate, and ensures that information such as health status, job transition, and intention to leave the nursing career are regularly monitored. Second, mortality data generated by the National Civil Registration are linked with the TNC database; confidentiality on personal data is strictly observed throughout.

This report presented details of study design and administration of the TNCS. We also provided details of weighting for statistical methods to be used with the TNCS data. We strongly recommended that any analyses should implement weights we provided in the report, or at least, should explore how weighting affects their conclusions. In this report, we found that Thai nurse were aging. About half of them, 48.5 %, were older than 44 years. The average age was 43.7 years old. However, this is partly due to that older nurses participated in the TNCS more than the younger. We then sought to get the average age based on the TNC database- it was 37 years. This was slightly younger when compared to what was reported in a study in the United States which estimated that by 2008 the average age of RNs was 45.4 years, with more than 40 % of the RN workforce were older than 50 years [10].

We found that 15.4 % of Thai RNs intended to leave nursing career someday during their employments. However, the percentage of intention to leave within the next 2 years was 4.3 %. By this proportion, based on the current RN population in Thailand, it is approximately 20,000 RNs who intended to leave their careers in the next two years. This could be an alarmingly high turnover rate.

## Conclusions

Health status of the current RNs were generally satisfactory. However, Thailand shares the same nursing crisis as many other countries, with a high proportion of aging nursing workforce, high rate of intention to leave nursing career, and low number of new entry nurses. Further studies are needed to investigate root causes of the crisis so that specific measures can be formulated to minimize the problem.

## Availability of data and materials

All original data were archived in a secure database server (http://www.thainursecohort.org). This server archived all study material and served as the portal for cohort members to respond to the survey questionnaire, view the survey summary results in a real-time manner, and participate in an electronic forum for information exchange among cohort members. Cohort members and other Thai researchers are encouraged to use the data for research purposes at no cost. To access data, researchers are required to submit an analysis plan proposal (APP) (via http://tncs.damus.in.th). The Data Archival for Maximize Utilization (DAMUS) Committee is responsible for reviewing and approving research requests. Researchers with an approved APP are required to provide regular progress updates until publication of their work. Currently, all study materials are in the Thai language; international researchers will need to contact the DAMUS Committee to collaborate with the TNCS and access the data.

## Abbreviations

AEC: ASEAN Economic Community
APP: analysis Plan Proposal
ASEAN: association of Southeast Asian Nations
DAMUS: data Archival for Maximize Utilization Committee
EQ-5D: EuroQol five-dimension questionnaire
IPAQ: international Physical Activity Questionnaire
JCQ: job Content Questionnaire short version
RN: registered nurse
SEA: South East Asia
TNC: Thailand Nursing and Midwifery Council
TNCS: Thai Nurse Cohort Study
